# Greater genetic risk for adult psychiatric diseases increases vulnerability to adverse outcome after preterm birth

**DOI:** 10.1038/s41598-021-90045-5

**Published:** 2021-06-01

**Authors:** Harriet Cullen, Saskia Selzam, Konstantina Dimitrakopoulou, Robert Plomin, A. David Edwards

**Affiliations:** 1 Centre for the Developing Brain, School of Biomedical Engineering and Imaging Sciences, King’s College London, London, SE1 7EH UK; 2 Department of Medical and Molecular Genetics, School of Basic and Medical Biosciences, King’s College London, London, SE1 9RT UK; 3 Institute of Psychiatry, Psychology and Neuroscience, King’s College London, London, SE5 8AF UK; 4 Translational Bioinformatics Platform, NIHR Biomedical Research Centre, Guy’s and St Thomas’ NHS Foundation Trust and King’s College London, London, SE1 9RT UK

**Keywords:** Genetics, Neuroscience

## Abstract

Preterm birth is an extreme environmental stress associated with an increased risk of later cognitive dysfunction and mental health problems. However, the extent to which preterm birth is modulated by genetic variation remains largely unclear. Here, we test for an interaction effect between psychiatric polygenic risk and gestational age at birth on cognition at age four. Our sample comprises 4934 unrelated individuals (2066 individuals born < 37 weeks, 918 born <  = 34 weeks). Genome-wide polygenic scores (GPS’s) were calculated for each individual for five different psychiatric pathologies: Schizophrenia, Bipolar Disorder, Major Depressive Disorder, Attention Deficit Hyperactivity Disorder and Autism Spectrum Disorder. Linear regression modelling was used to estimate the interaction effect between psychiatric GPS and gestational age at birth (GA) on cognitive outcome for the five psychiatric disorders. We found a significant interaction effect between Schizophrenia GPS and GA (*β* = 0.038 ± 0.013, *p* = 6.85 × 10^–3^) and Bipolar Disorder GPS and GA (*β* = 0.038 ± 0.014, *p* = 6.61 × 10^–3^) on cognitive outcome. Individuals with greater genetic risk for Schizophrenia or Bipolar Disorder are more vulnerable to the adverse effects of birth at early gestational age on brain development, as assessed by cognition at age four. Better understanding of gene-environment interactions will inform more effective risk-reducing interventions for this vulnerable population.

## Introduction

Preterm birth is an extreme perinatal stress and is associated with a significantly increased risk of later cognitive dysfunction and psychiatric disease. Preterm birth is common, accounting for around 11% of all births and is a leading cause of infant mortality and morbidity worldwide^1^. Preterm infants have higher rates of cognitive impairment, cerebral palsy, autism spectrum disorders and psychiatric disease^2–5^, not all of which can be explained by the degree of prematurity and severity of clinical problems around the time of birth.

Magnetic resonance imaging studies indicate that preterm brain injury is a multifactorial disorder which encompasses alterations to cortical and subcortical gray matter and white matter. These studies have contributed to our understanding of the pathophysiology underlying neurodevelopmental disability and cognitive impairments in the preterm population, however, a great deal remains unknown. Specifically, the extent to which genetic factors play a role in the variable cognitive outcomes of preterm infants and the possible interaction between genetic risk and the environmental exposure to preterm birth has not been examined in detail. Here, we explore whether common genetic variation is important in determining an individual’s susceptibility to the environmental stress of prematurity and thus plays a role in the diverse neurodevelopmental and cognitive outcomes we observe in preterm infants.

Research into the genetics of psychiatric disease has shown it to be polygenic; psychiatric disorders are influenced by many genetic variants, each of small effect^6^. We therefore anticipate that gene-environment interactions involving these complex pathologies will be most effectively studied by looking at multiple genetic variants rather than focussing on a single genetic locus. As samples sizes have grown, genome-wide association studies (GWAS) have become increasingly informative, allowing detection of small effects of single nucleotide polymorphisms (SNPs). Using the summary statistics from these studies it is possible to generate individual-specific genotypic scores to predict phenotypic variance, genome-wide polygenic scores (GPS). A genome-wide polygenic score, which is an aggregate of trait-related effect sizes of SNPs across the genome in independent samples can be used to test the predictive power of multiple genetic variants simultaneously. Genome-wide polygenic scores provide a useful approach to exploring gene-environment interactions in complex traits.

In previous work, we hypothesised that genes associated with psychiatric disease might increase vulnerability to abnormal brain development in infants subjected to the environmental stress of prematurity^7^. Under this notion, individuals with a greater polygenic risk for psychiatric disease, when born preterm, would be more likely to suffer adverse developmental consequences. We showed that in a cohort of preterm infants, psychiatric GPS was negatively associated with lentiform volume; genetic predictors of neuropsychiatric disease increase vulnerability to abnormal lentiform development after perinatal stress.

We now extend this work by looking at the possible influence of genome-wide polygenic risk for psychiatric disease in preterm individuals, as measured by their cognitive abilities in early childhood. In a large cohort of unrelated twins, we test for a possible gene-environment interaction between the GPS’s for five different psychiatric disorders (Autism Spectrum Disorder (ASD), Attention Deficit Hyperactivity Disorder (ADHD), Bipolar Disorder, Major Depressive Disorder and Schizophrenia) and gestational age at birth on cognition at age four.

## Results

Linear regression modelling was used to test for a possible gene-environment interaction between the GPS’s for five different psychiatric disorders (ASD, ADHD, Bipolar Disorder, Major Depressive Disorder and Schizophrenia) and gestational age at birth on cognition at age four. Genome-wide polygenic scores were adjusted for the first five ancestry principle components, genotype chip and plate and each regression model included the covariates sex and socio-economic status (SES) (Eq. (1)).

Genotype, phenotype and covariate information was available for 4934 individuals (2594 females and 2340 males). Of these 2066 were born before 37 weeks completed gestation and 918 were born at or below 34 weeks completed gestation. A table showing the number of individuals at different gestational age cut-offs is included in the Supplementary Information (Supplementary Table S2).

Results indicated a significant interaction between psychiatric genetic risk and gestational age at birth for two of the five pathologies investigated (Table 1) after Bonferroni adjustment for multiple testing (p < 0.05/5 = 0.01). There was evidence for an interaction effect for gestational age at birth and Schizophrenia genetic risk (*β*_*3*_ = *0.038, se* = *0.013,* p_3_ = 6.85 × 10^–3^*)* and an interaction effect for gestational age at birth and Bipolar genetic risk (*β*_*3*_ = 0.038, se = 0.014, p_3_ = 6.61 × 10^–3^). There was no evidence for an interaction effect between gestational age at birth and genetic risk for Autism Spectrum Disorder, ADHD or Major Depressive Disorder. For both Schizophrenia and Bipolar Disorder, the effect of gestational age at birth on cognitive outcome was greater for those individuals with a larger psychiatric genetic risk burden. Results for the full regression model for all five psychiatric pathologies are given in Supplementary Table S3.

**Table 1:** Gene-environment interaction between genetic risk for psychiatric disease and gestational age at birth on cognition at four.

Psychiatric GPS	*GPS*_*psych*_ × *GA*		
	*β*_3_	*SE*	*p*_3_
ADHD	− 0.015	0.016	0.302
ASD	− 0.003	0.015	0.846
**Bipolar Disorder**	**0.038**	**0.014**	**6.61 × 10**^**–3**^
Major Depressive Disorder	0.001	0.014	0.958
**Schizophrenia**	**0.038**	**0.013**	**6.85 × 10**^**–3**^

Results exploring the effect of the interaction term GPS_psych_ × GA on cognitive outcome in 4934 unrelated individuals. *β*_*3*_ is the estimate of the effect size of the interaction term *GPS*_*psych*_ × *GA*, *SE* is the standard error of the effect size and *p*_3_ is the corresponding p-value. Bonferroni correction was used to adjust for multiple testing and p-values < 0.01 (0.05/5) were considered statistically significant. Significant results are indicated in bold. Beta regression coefficients and associated p-values for all terms in the regression model are detailed in Supplementary Table S3 in the Supplementary Information.

Removing the covariate socio-economic status from the model did not markedly alter the results. There remained a significant interaction effect of gestational age at birth and Schizophrenia genetic risk (*β* = *0.038, se* = *0.013,* p = 7.07 × 10^–3^) and gestational age at birth and Bipolar genetic risk (*β* = *0.040, se* = *0.014,* p = 4.60 × 10^–3^) on cognitive outcome at four*.* Further, there was no evidence for an association of cognition with twin birth order and the addition of this as a covariate to our model did not significantly alter the results. There remained a significant interaction effect of gestational age at birth and Schizophrenia genetic risk (*β* = *0.038, se* = *0.014,* p = 6.75 × 10^–3^) and gestational age at birth and Bipolar genetic risk (*β* = *0.039, se* = *0.014,* p = 6.00 × 10^–3^) on cognitive outcome at four.

In addition to our linear regression analysis, considering gestational age and psychiatric genetic risk as continuous variables, we compared the effect of gestational age at birth on cognition for those individuals in the highest and lowest quintiles of the Schizophrenia and Bipolar Disorder GPS distributions. For this analysis we selected only those individuals born at 34 weeks gestation or less and compared them with term-born individuals (> = 37 weeks).

**Figure 1:**
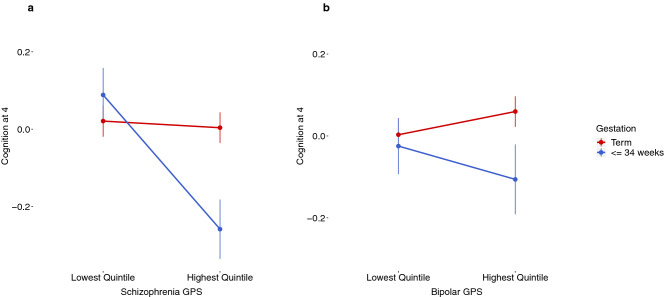
(**a**) Mean cognitive score at age four corrected for sex and SES by Schizophrenia GPS and gestational age for individuals falling in the highest and lowest 20% of the distribution of Schizophrenia GPS. There was evidence for an interaction effect (F(1506) = 8.03, p = 4.65 × 10^–3^); (**b**) Mean cognitive score at age 4 corrected for sex and SES by Bipolar disorder GPS and gestational age for individuals falling in the highest and lowest 20% of the distribution of the Bipolar Disorder GPS. No statistically significant interaction effect was found (F(1530) = 1.55 P = 0.213), *GPS*: genome-wide polygenic score.

Figure 1a compares the mean cognitive scores at age four corrected for sex and socio-economic status for both term-born individuals and individuals born at <  = 34 weeks in the highest and lowest quintiles of the Schizophrenia GPS distribution. Two-way ANOVA analysis confirms a statistically significant interaction between gestational age at birth and level of Schizophrenia risk on cognition (F(1506) = 8.03, p = 4.65 × 10^–3^). The adverse effect of birth at earlier gestational age (< = 34 weeks) on cognition is greater for individuals with a higher genetic risk burden for Schizophrenia than it is for those with the lowest risk (Fig. 1a and Supplementary Table S4).

Figure 1b compares the mean cognitive scores at age four, corrected for both sex and socio-economic status for both term-born individuals and individuals born at 34 weeks gestation or less in the highest and lowest quintiles of the Bipolar Disorder GPS distribution. In this case two-way ANOVA analysis did not indicate a significant interaction effect between gestational age at birth and level of Bipolar Disorder genetic risk on cognition (F(1530) = 1.55 p = 0.213). Although differences are observed, unlike Schizophrenia, the adverse effect of birth at early gestational age (< = 34 weeks) on cognition was not significantly different when comparing those individuals at highest and lowest risk of Bipolar Disorder (Fig. 1b and Supplementary Table S5).

The results of comparable analysis for ASD, ADHD and Major Depressive Disorder are presented in Supplementary Table S6. No significant interaction effects were observed for ASD, ADHD or Major Depressive Disorder, consistent with the linear regression analysis. In addition, a sensitivity analysis for the Schizophrenia ANOVA result is presented in the Supplementary Information exploring the effect of varying the gestational age cut-off (Supplementary Methods and Results and Supplementary Tables S7a,b).

Finally, our analysis confirmed that cognitive outcome at four shows a positive association with gestational age in our cohort (*β* = *0.039*, *se* = *0.014,* p = 5.5 × 10^–3^) including sex and socio-economic status as covariates. None of the psychiatric genetic risk scores showed a significant association with cognition at age four (Supplementary Methods and Results and Supplementary Table S10), consistent with previous work in the literature^8^.

## Discussion

In this work we tested for a possible interaction effect between psychiatric polygenic risk for five different psychiatric disorders and gestational age at birth on cognition at age four. We identified a significant interaction effect between genetic risk for both Bipolar Disorder and gestational age at birth and genetic risk for Schizophrenia and gestational age at birth. Those individuals with a greater genetic risk burden for either Bipolar Disorder or Schizophrenia appear to be more vulnerable to the adverse effects of birth at earlier gestational age on cognitive outcome. No significant interaction effect was observed for genetic risk for ASD, ADHD or Major Depressive Disorder and gestational age at birth.

The similar results obtained for both Bipolar Disorder and Schizophrenia are consistent with evidence for shared genetic aetiology for these disorders. In our cohort there is a significant correlation between genetic risk for Bipolar Disorder and Schizophrenia (r = 0.266, p < 2.2 × 10^–16^, Supplementary Table S8) which accords with results in the literature. In a large cohort, examining several disorders of the brain including psychiatric pathologies, the most robust correlation was observed between genetic risk for Schizophrenia and Bipolar Disorder^9^ (correlation r = 0.6808, p = 2.13 × 10^–230^).

The observed correlation between genetic risk for Bipolar Disorder and Schizophrenia in our cohort motivated us to undertake an exploratory analysis looking at whether the interaction terms for Schizophrenia and Bipolar Disorder had an independent contribution to the regression model for cognition. We did this using stepwise linear regression and the final model included interaction terms for both pathologies (Supplementary Methods and Results and Supplementary Table S9).

Many studies in the literature have demonstrated an association between preterm birth and poorer cognitive outcome^10–12^. This trend was replicated in our smaller dataset where we observed a positive association between cognition at four and gestational age at birth. Despite this consistent observation there remains a great deal of variability in the neurodevelopmental outcomes of preterm infants and much that is not understood about the underlying pathophysiology. The extent to which common genetic variation may play a role in understanding why some infants appear to be at greater risk from the environmental stress of prematurity remains largely unexplored. This work starts to address this question by asking if the effect of prematurity on cognitive outcome is dependent on genotype, and specifically psychiatric risk burden. In doing so we hope to be able to improve assessment of an individual’s risk of cognitive impairment and better understand the pathophysiology underling the diverse neurodevelopmental outcomes observed in preterm infants. This will ultimately enable more effective identification of those infants at greatest risk of poorer outcomes and allow us to target therapeutic interventions appropriately.

The focus on genetic risk for psychiatric pathologies was motivated by previous work showing abnormal lentiform development in preterm infants with a greater genetic risk burden for psychiatric disease^7^. Furthermore, preterm birth is known to confer an increased risk of psychopathology. Preterm birth is associated with an increased risk of psychiatric hospitalization in adulthood across a range of psychiatric disorders^5^; the greatest increased risk is observed for Bipolar Disorder, followed by non-affective psychosis, which includes Schizophrenia and Schizoaffective Disorder.

Gene–environment interactions are the result of individuals responding differently to environmental stimuli, depending on their genotype. Many studies have explored gene-environment interactions for psychiatric pathologies^13^ and much of this work has focussed on understanding how stressful life events interact with genotype to affect risk of psychiatric conditions. The work of Capsi and colleagues^14^, which focused on the interaction between a functional candidate polymorphism in the serotonin transporter gene (5-HTT) and stressful life events, provides an early example of such a study. The authors showed that the level of depressive symptoms experienced by individuals in response to stressful life events was dependant on the allele they carried at this polymorphism.

Whilst early studies largely focussed on single polymorphisms, evidence suggests that a polygenic approach is likely to be more fruitful in understanding how genetic risk for psychiatric pathologies may increase susceptibility to environmental risk factors. More recent studies frequently investigate gene-environment interactions on a genome-wide scale, leveraging results from genome-wide association studies to construct polygenic scores. It is hoped that this approach could reveal interactions that may have gone undetected using candidate SNP analysis.

Gene-environment interaction studies exploring Schizophrenia have largely focussed on stressful life events^15^ and early life adversity^16,17^ with a few studies exploring perinatal factors. Studies with a perinatal focus include work indicating a significant interaction between maternal CMV infection and a polymorphism in the CTNNA3 gene on schizophrenia risk^18^ and work indicating that low birth weight predicts poorer academic and physical performance in individuals at high risk of Schizophrenia but not for those at low risk^19^.

Studies exploring Bipolar Disorder have followed similar themes to those for Schizophrenia focussing on environmental exposure to stressful life events^13^. The majority of work on Bipolar Disorder has been using candidate genes and to the best of our knowledge there have been no previous studies using summary statistics for Bipolar Disorder from the Psychiatric Genomics Consortium.

### Limitations

Preterm birth itself may be a genetically inherited trait. However, it is difficult to know the extent to which this could contribute to confounding in this work. Despite evidence for a modest contribution of genetics to the risk of preterm birth, to-date, there exist few robust genetic associations identified through genome-wide association studies for prematurity. A large recent study identified two intergenic loci associated with preterm birth but failed to replicate these results^20^.

Preterm birth is complex with heterogenous aetiology. Data that would allow us to distinguish between different aetiologies such as information on maternal infection or maternal pathology were not available in this study. It is possible that genetic risk alleles that confer an increased risk of Schizophrenia or Bipolar Disorder might also confer an increased risk of pathologies associated with preterm birth (e.g. pre-eclampsia, cervical insufficiency). Whilst we believe that such shared genetic aetiology, if present, is unlikely to fully explain our results, we cannot exclude the possibility that it could introduce confounding.

We have explored the possibility that genetic risk for psychiatric disease is associated with gestational age at birth within our cohort. For the five psychiatric pathologies examined in this study we found no significant association between genetic risk for psychiatric disease and gestational age at birth (Supplementary Methods and Results and Supplementary Table S11). Our result corroborates results in the literature: no association was found between genetic risk for ASD, ADHD or Schizophrenia and prematurity in a cohort of 7921 mothers and children from the Avon Longitudinal Study of Parents and Children^21^. That said, we cannot exclude the possibility that we are underpowered to detect such an association.

Environmental factors might additionally convey their influence through epigenetic mechanisms. There is growing evidence to suggest a role of DNA methylation in mediating the effects of preterm birth on future outcomes. For example, recent work in a cohort of 3648 newborns identified numerous differentially methylated CpG sites in relation to gestational age at birth^22^. Further exploration of epigenetic modification associated with prematurity would be a relevant future study and one we hope to undertake using the Developing Human Connectome Cohort.

Finally, this study focused on cognitive outcome at age four. We hypothesised that the interaction between genetic risk for psychiatric disease and gestational age at birth would explain a significant fraction of the variability in cognitive outcome at age four. Work looking at preterm outcomes has shown stability of impairment in early childhood and confirmed that the most commonly observed impairment is in developmental or cognitive function^2^. In future work we hope to look at whether or not our results extend to cognitive outcome at school age (7 and 12). As children grow older, smaller differences in ability might be easier to measure but they will similarly be subjected to a larger diversity of additional environmental exposures.

### Summary

In summary, individuals with greater genetic risk for either Schizophrenia or Bipolar Disorder were shown to be more vulnerable to the negative effects of birth at earlier gestational age on cognitive outcome. The pathophysiology underlying preterm neurodevelopmental impairment may therefore be vulnerable to genetic variability associated with adult psychiatric disease.

## Materials and methods

### Subjects

Participants were drawn from the Twins Early Development Study (TEDS). Between 1994 and 1996 TEDS recruited over 15,000 twin pairs born in England and Wales, who have been assessed in multiple waves across their development up until the present date. Roughly 10,000 twin pairs still actively contribute to TEDS, providing genetic, cognitive, psychological and behavioural data. TEDS participants and their families are representative of families in the UK^23^. Written informed consent was obtained from parents prior to data collection. Project approval was granted by King’s College London’s Research Ethics Committee for the Institute of Psychiatry, Psychology and Neuroscience (PNM/09/10-104) and the research was performed in compliance with the Declaration of Helsinki.

This study included 4934 unrelated individuals of European ancestry from the TEDS cohort. The dataset was subject to standard exclusions. This included exclusion of individuals with serious medical conditions which would likely affect their ability to take part in the TEDS assessments or conditions known to be associated with significant cognitive impairment. It also included exclusion of individuals in whom extreme circumstances were identified in the perinatal period such as prolonged hospital admission following birth. An initial filtering was then undertaken to select a cohort of unrelated individuals; this involved selection of all the unpaired twins plus one twin from each genotyped pair. Individuals were retained that had genotyping for the GPS analysis, data for cognitive outcome at age four, recorded gestational age at birth and recorded parental socio-economic status.

The final sample comprised 4934 individuals. Of these, 1858 were from monozygotic (MZ) twin pairs and 3076 were from dizygotic (DZ) twin pairs (1596 same-sex DZ pairs and 1480 opposite-sex DZ pairs). 2066 of the 4934 individuals were born at less than 37 weeks completed gestation and 918 were born at or below 34 weeks gestation.

### Genotyping

Genotyping was undertaken at two separate timepoints with two different genotyping platforms. 1744 of the 4934 individuals were genotyped on the Affymetrix GeneChip 6.0 SNP array at Affymetrix, Santa Clara (California, USA) using buccal cell DNA samples. Genotypes were generated at the Wellcome Trust Sanger Institute (Hinxton, UK) as part of the Wellcome Trust Case Control Consortium 2 (https://www.wtccc.org.uk/ccc2/). The remaining 3190 individuals were genotyped on HumanOmniExpressExome-8v1.2 arrays at the Molecular Genetics Laboratories of the Medical Research Council Social, Genetic Developmental Psychiatry Centre, using DNA that was extracted from saliva samples.

After quality control, 635,269 SNPs remained for Affymetrix GeneChip 6.0 genotypes, and 559,772 SNPs for HumanOmniExpressExome genotypes. Genotypes from the two platforms were separately phased using EAGLE2^24^, and imputed into the Haplotype Reference Consortium (release 1.1) through the Sanger Imputation Service before merging genotype data from both platforms (for more details see Selzem et al.^25^).

The final data contained 7,363,646 genotyped or well imputed SNPs. Principal component analysis was performed on a subset of 39,353 common (MAF > 5%), perfectly-imputed (info = 1) autosomal SNPs, after stringent pruning to remove markers in linkage disequilibrium (r^2^ > 0.1) and excluding high linkage disequilibrium genomic regions.

### Phenotypic data

#### Cognition aged four

The standardised cognitive ability measure at four was a composite of ‘Parent Report of Children’s Abilities’ (PARCA) including both a parent-reported and parent-administered element and an assessment of verbal and non-verbal ability. The parent administered assessment included an odd-one-out task, design drawing task and puzzle task and the parent-reported assessment examined conceptual knowledge. Non-verbal ability was assessed with a 48-item vocabulary test and verbal ability with a grammar assessment which looked at length of sentences and correct use of both ‘-est’ words and ‘but’. The parent administered assessments have been validated in a subsample^26^. The cognitive ability measure was computed as the mean of the four standardised elements.

#### Family socio-economic status (SES)

Socio-economic status is a composite of maternal age at birth of eldest child, the mean score of maternal and paternal highest education level, as well as the respondent’s (mother or father) occupation, administered by the Standard Occupational Classification 2000 (Office for National Statistics, 2000) when the child was two, which was the first age of contact.

### Genome-wide polygenic scores

We calculated genome-wide polygenic scores, which are the SNP effect-size weighted sums of the number of trait-associated alleles. The SNP weights were derived from summary statistics for the largest GWA studies available for five psychiatric disorders at time of computation (see Supplementary Table S1).

The genome-wide polygenic scores were calculated using the software LDpred^27^ which re-weights the SNP effect sizes based on a prior on the effect size and the LD in the sample. To ease computational demands, we selected variants with an info score of one, resulting in 515,000 SNPs used for analysis. In this work, we applied a prior on the fraction of causal markers of one for all analyses, based on the assumption that all genetic markers contribute to trait development. All polygenic scores were statistically adjusted for the first five ancestry principal components, chip and plate using the regression method and were z-standardized (mean = 0, SD = 1).

### Statistical analysis

Linear regression modelling was used to estimate the effect of the gene-environment interaction (GPS_psych_ × GA) on cognitive outcome at age four, for five different psychiatric pathologies (ADHD, ASD, Bipolar Disorder, Major Depressive Disorder and Schizophrenia) separately (Eq. (1)). Prior to inclusion in the model, the genome-wide polygenic scores were adjusted for ancestry, genotype chip and plate. Each model included the covariates sex and socio-economic status. Evidence suggests socio-economic status is an important predictor of cognitive outcome in both children and adults and that preterm infants may be particularly vulnerable to the effects^28^. Interaction terms for psychiatric genome-wide polygenic score (GPS_psych_) with sex and socio-economic status as well as interaction terms for gestational age at birth (GA) with sex and socio-economic status were included to properly control for possible confounding effects^29^.

1 $${Cognition}_{age4}= {\beta }_{0 }+{\beta }_{1} {GPS}_{psych}+{\beta }_{2}GA+{{\varvec{\beta}}}_{3}\left({{\varvec{G}}{\varvec{P}}{\varvec{S}}}_{{\varvec{p}}{\varvec{s}}{\varvec{y}}{\varvec{c}}{\varvec{h}}\boldsymbol{ }}\times {\varvec{G}}{\varvec{A}}\right) +{\beta }_{4}\left(sex\right)+{\beta }_{5}\left(SES\right)+{\beta }_{6}\left({GPS}_{psych }\times sex\right)+{\beta }_{7}\left({GPS}_{psych}\times SES\right)+{\beta }_{8}\left(GA\times sex\right)+{\beta }_{9}\left(GA\times SES\right)+ \varepsilon$$

The aim of this analysis was to investigate the effect of the interaction term GPS_psych_ × GA on cognitive outcome. We therefore explored whether the estimate of the coefficient *β*_*3*_ of the interaction term deviated significantly from zero. P-values lower than the significance level α = 0.05/5 = 0.01 were considered significant to account for the family-wise error rate using the Bonferroni method, correcting for the five psychiatric pathologies explored. Calculations were implemented with “lm” function in R (https://www.r-project.org/). Standard errors for the beta regression coefficients were estimated using the bootstrap technique with 10,000 bootstrap samples with random sampling of individuals with replacement.

As a further exploration of our data, we looked at the extremes of the data undertaking a two-way analysis of variance (ANOVA) comparing mean cognition between the extreme quintiles of polygenic risk for individuals born at or below 34 weeks gestation and term-born (> = 37 weeks) individuals for the psychiatric pathologies that yielded significant results in our initial analysis. Similar analysis for the non-significant pathologies is presented in the Supplementary Information (Supplementary Table S6).

In addition to our interaction analysis we sought to confirm that gestational age in our cohort predicted cognitive outcome at four. This was investigated using linear regression modelling, including both sex and socio-economic status as covariates (Eq. (2)).

2 $${Cognition}_{age4}= {\beta }_{0 }+{\beta }_{1} GA+{\beta }_{2}\left(sex\right)+{\beta }_{3}\left(SES\right)+ \varepsilon$$

For completeness we also explored a possible association between cognition at four and genetic risk for each of the five psychiatric pathologies examined, details can be found in the Supplementary Analysis (Supplementary Methods and Results and Supplementary Table S10).

### Ethics

Written informed consent was obtained from parents prior to data collection. Project approval was granted by King’s College London’s Research Ethics Committee for the Institute of Psychiatry, Psychology and Neuroscience (PNM/09/10-104). The research was performed in compliance with the Declaration of Helsinki.

## Supplementary Information

Supplementary Information.

## Data Availability

The Twins Early Development Study dataset is a controlled-access dataset that is accessible through collaboration with core TEDS researchers. A full explanation of the data access policy can be found at http://www.teds.ac.uk/research/collaborators-and-data/teds-data-access-policy.
