# Supplementary material for: Greater genetic risk for adult psychiatric diseases increases vulnerability to adverse outcome after preterm birth
Source: Sci Rep. 2021 Jun 1;11:11443. doi: 10.1038/s41598-021-90045-5 (PMC8169748; doi:10.1038/s41598-021-90045-5)
Supplement: Supplementary file 1 — Supplementary Information. [file 41598_2021_90045_MOESM1_ESM.docx]

Supplementary Information for:

**Greater genetic risk for adult psychiatric diseases increases vulnerability to adverse outcome after preterm birth**

Harriet Cullen^*1,2^, Saskia Selzam^3^, Konstantina Dimitrakopoulou^4^, Robert Plomin^3^, A David Edwards^1^

^1^ Centre for the Developing Brain, School of Biomedical Engineering and Imaging Sciences, King’s College London, London SE1 7EH, United Kingdom

^2^ Department of Medical and Molecular Genetics, School of Basic and Medical Biosciences, King’s College London, London SE1 9RT, United Kingdom

^3^ Institute of Psychiatry, Psychology and Neuroscience, King’s College London, London SE5 8AF, United Kingdom

^4^ Translational Bioinformatics Platform, NIHR Biomedical Research Centre, Guy's and St

Thomas' NHS Foundation Trust and King's College London, London SE1 9RT, United Kingdom

**Correspondence author:** Harriet Cullen

**Email:** harriet.cullen@kcl.ac.uk

**This file includes:**

**Supplementary Text**: Supplementary Methods and Results

1. Stepwise Linear Regression
2. Association of Psychiatric Genetic Risk Scores with Cognitive Outcome at Four
3. Association of Psychiatric Genetic Risk Scores with Gestational Age at Birth
4. Sensitivity Analysis for ANOVA Result for Schizophrenia Genetic Risk and Gestational Age

**Supplementary Tables**: Supplementary Table S1-S11

**Supplementary References**

**Supplementary Text**

**Supplementary Methods and Results: Stepwise linear regression**

There is a strong degree of correlation between several of the psychiatric genetic risk scores in our cohort (Supplementary Table S8). For the two pathologies for which we obtained significant results, Schizophrenia and Bipolar Disorder, there is a significant correlation between the genome-wide polygenic scores (*r = 0.266, p < 2.2x10^-16^*) for the 4934 individuals studied. To try and better determine whether the interaction terms for Schizophrenia and gestational age and Bipolar Disorder and gestational age have an independent contribution to the regression model for cognition we performed stepwise linear regression using the ‘step’ function included in the ‘stats’ package in R.

A base model for cognitive outcome was constructed that included both Schizophrenia and Bipolar genetic risk (corrected for ancestry, batch, and chip) and the covariates gestational age at birth, sex, and socio-economic status (SES). Stepwise linear regression was then performed using ‘both’ directions so that the interaction terms for Schizophrenia with gestational age and Bipolar Disorder with gestational age were either added or dropped depending on how they improved the Akaike information criterion (AIC) of the model. All secondary interactions between variables were also included to control for potential confounding^1^. The process was repeated until no improvement in AIC could be made.

Base model:

${Cognition}_{age4}= \beta_{0}+\beta_{1} {GPS}_{SCZ}+{\beta_{2}GPS}_{Bipolar}+ \beta_{3}GA +\beta_{4}\left( sex \right)+\beta_{5}\left( SES \right)+ \varepsilon$

The final model generated by the stepwise linear regression included the genetic risk x gestational age interaction terms for both Schizophrenia and Bipolar Disorder. Both the interaction terms were nominally significant. The results are indicated in Supplementary Table S9.

Final model:

${Cognition}_{age4}= \beta_{0}+\beta_{1} {GPS}_{SCZ}+{\beta_{2}GPS}_{Bipolar}+ \beta_{3}GA +\beta_{4}\left( sex \right)+\beta_{5}\left( SES \right)+\beta_{6}\left( {GPS}_{SCZ}\times GA \right)+ \beta_{7}\left( {GPS}_{Bipolar}\times GA \right)+\beta_{8}\left( GA\times sex \right)+\varepsilon$

**Supplementary Methods and Results: Association of Psychiatric Genetic Risk Scores with Cognitive Outcome at Four**

Additional analysis was undertaken to explore a possible association of the five genome-wide polygenic scores for ADHD, ASD, Bipolar Disorder, Major Depressive Disorder and Schizophrenia with cognitive outcome at age four. Genome-wide polygenic scores were adjusted for the first five ancestry principle components, genotype chip and plate and each regression model included sex and socio-economic status as covariates.

$${Cognition}_{age4}= \beta_{0}+\beta_{1} {(GPS}_{psych})+\beta_{2}\left( sex \right)+\beta_{3}\left( SES \right)+ \varepsilon$$

Results indicated there was no statistically significant association between any of the psychiatric genome-wide polygenic scores and cognition at age four. Results are given in Supplementary Table S10.

**Supplementary Methods and Results: Association of Psychiatric Genetic Risk Scores with Gestational Age at Birth**

Additional analysis was undertaken to explore a possible association of the five genome-wide polygenic scores for ADHD, ASD, Bipolar Disorder, Major Depressive Disorder and Schizophrenia with gestational age at birth. Genome-wide polygenic scores were adjusted for the first five ancestry principle components, genotype chip and plate and each regression model included sex as a covariate.

$${GPS}_{psych}= \beta_{0}+\beta_{1} (GA)+\beta_{2}\left( sex \right)+ \varepsilon$$

We found no statistically significant association between the psychiatric GPS’s and gestational age at birth. Our data suggested a modest negative association between the genetic risk for ADHD and gestational age at birth *(β = -0.028, SE=0.014 p = 0.055),* however this was not statistically significant. Results are given in Supplementary Table S11.

**Supplementary Methods and Results: Sensitivity Analysis for ANOVA result for Schizophrenia genetic risk and gestational age.**

Here we present a sensitivity analysis of the Schizophrenia ANOVA results comparing the effect of gestational age at birth on cognition for those individuals in the highest and lowest quintiles of the Schizophrenia GPS distribution. We explore how the interaction effect is influenced by varying the gestational age cut-off used to define the subsamples.

In the first part of the analysis, we compare different subsets of the preterm population to term-born individuals for the top and bottom quintiles of the Schizophrenia GPS distribution. We relax the gestational age threshold used in the main manuscript (<=34 weeks) and instead compare individuals born at <= 35 weeks with term-born individuals. We find that there remains a significant interaction effect between gestational age at birth and Schizophrenia genetic risk on cognitive outcome (*F(1698) = 5.791, p = 0.016*). Similarly, if we make the preterm cut-off more stringent, we continue to observe a significant interaction effect when comparing term-born individuals with individuals born at <= 33 weeks (*F(1357) = 6.703, p = 9.73x10^-3^* ) and individuals born at <=32 weeks (*F(1291) = 4.403, p=0.036*). These results are presented in Supplementary Table S7a. Extending this analysis to Bipolar Disorder we find the interaction effect remains non-significant at these additional gestational age cut-offs.

We also take a different approach and rather than selecting a subset of the preterm infants, we instead split the cohort at a specific gestational age and then compare all individuals in the top and bottom quintiles of the risk score distribution. Splitting the cohort at a gestational age of 37 weeks, including only those individuals in the top and bottom quintiles of the risk score distribution, we do not see a significant interaction between prematurity and Schizophrenia risk on cognitive outcome (*F (1968) = 1.348, p= 0.246*). However, if we reduce the gestational age cut-off and instead split the cohort at a gestational age of 36, 35, 34 or 33 weeks, we find a significant interaction effect at all of these cut-offs. These results are presented in Supplementary Table 7b. Repeating this analysis for Bipolar Disorder we do not see a significant interaction effect at any gestational age split.

These additional results would suggest that the negative effect of a greater genetic risk burden for Schizophrenia is detectable in our cohort in individuals born below 36 weeks completed gestation.

**Supplementary Table S1. Genome-Wide Association Studies Used to Compute the Genome-Wide Polygenic Scores in this Study.**

| **Psychiatric Pathology** | **Reference** | **Year Published** | **Cases** | **Controls** | **Sample Size** |
| --- | --- | --- | --- | --- | --- |
| ADHD | Demontis et al.^2^ | 2019 | 20183 | 35,191 | 55374 |
| ASD | Grove et al.^3^ | 2019 | ﻿18381 | ﻿27969 | 46350 |
| Bipolar Disorder | Sklar et al.^4^ | 2011 | ﻿ ﻿7481 | ﻿ ﻿9250 | 16731 |
| Major Depressive Disorder | Wray et al.^5^ | 2018 | 59851 | 113154 | 173005 |
| Schizophrenia | Pardiñas et al.^6^ | 2018 | 40675 | 64643 | 105318 |

## ***Supplementary Table S1***. Publicly available Psychiatric Genomics Consortium Genome-wide association studies used for the computation of the genome-wide polygenic scores in this study. ADHD – Attention Deficit Hyperactivity Disorder, ASD – Autism Spectrum Disorder.

**Supplementary Table S2. Table detailing the number of individuals in the TEDS cohort used for this analysis at different gestational age cut-offs.**

| **Gestational age cut-off** | **Number of Individuals** |
| --- | --- |
| >= 37.0 weeks | 2868 |
| < 37.0 weeks | 2066 |
| <= 34.0 weeks | 918 |
| < 32.0 weeks | 193 |

**Supplementary Table S3. Beta regression coefficients and associated p-values for all terms in the linear regression model (Equation 1) for each of the psychiatric pathologies.**

*Equation (1):*

${Cognition}_{age4}= \beta_{0}+\beta_{1} {GPS}_{psych}+\beta_{2}GA+\boldsymbol{\beta}_{\boldsymbol{3}}\left( \boldsymbol{GPS}_{\boldsymbol{psych}}\boldsymbol{\times GA} \right) +\beta_{4}\left( sex \right)+\beta_{5}\left( SES \right)+\beta_{6}\left( {GPS}_{psych}\times sex \right)+\beta_{7}\left( {GPS}_{psych}\times SES \right)+\beta_{8}\left( GA\times sex \right)+\beta_{9}\left( GA\times SES \right)+ \varepsilon$

|  | *GPS_psych_* | | *GA* | | ***GPS_psych_ x GA*** | | *Sex* | | *SES* | |
| --- | --- | --- | --- | --- | --- | --- | --- | --- | --- | --- |
|  | *β_1_* | p_1_ | *β_2_* | p_2_ | ***β_3_*** | **p_3_** | *β_4_* | p_4_ | *β_5_* | p_5_ |
| Schizophrenia | -0.023 | 0.229 | 0.008 | 0.672 | **0.038** | **6.85x10^-3^** | -0.173 | 7.12x10^-10^ | 0.159 | < 2x10^-16^ |
| Bipolar Dis. | -0.012 | 0.527 | 0.008 | 0.689 | **0.038** | **6.61x10^-3^** | -0.172 | 8.07x10^-10^ | 0.159 | < 2x10^-16^ |
| ASD | -0.007 | 0.717 | 0.008 | 0.691 | **-0.003** | **0.846** | -0.173 | 7.32x10^-10^ | 0.159 | < 2x10^-16^ |
| ADHD | -0.002 | 0.906 | 0.008 | 0.665 | **-0.015** | **0.302** | -0.171 | 1.11x10^-9^ | 0.159 | < 2x10^-16^ |
| MDD | -0.023 | 0.239 | 0.008 | 0.696 | **0.001** | **0.958** | -0.172 | 9.45x10^-10^ | 0.157 | < 2x10^-16^ |

|  | *GPS_psych_ x sex* | | *GPS_psych_ x SES* | | *GA x sex* | | *GA x SES* | |
| --- | --- | --- | --- | --- | --- | --- | --- | --- |
|  | *β_6_* | p_6_ | *β_7_* | p_7_ | *β_8_* | p_8_ | *β_9_* | p_9_ |
| Schizophrenia | 0.010 | 0.725 | -0.007 | 0.627 | 0.066 | 0.019 | -0.009 | 0.523 |
| Bipolar Dis. | 0.026 | 0.351 | 0.007 | 0.634 | 0.065 | 0.020 | -0.009 | 0.499 |
| ASD | 0.031 | 0.273 | 0.002 | 0.894 | 0.064 | 0.022 | -0.009 | 0.536 |
| ADHD | 0.006 | 0.822 | 0.027 | 0.057 | 0.065 | 0.021 | -0.010 | 0.494 |
| MDD | -0.001 | 0.967 | 0.008 | 0.556 | 0.064 | 0.022 | -0.009 | 0.536 |

**Supplementary Table S3**. Beta regression coefficients and associated p-values for all terms in the linear regression model (Equation 1) for each of the five psychiatric pathologies. All continuous variables have been scaled to have zero mean and standard deviation of 1. Sex is a categorical variable with levels 0 and 1 for females and males respectively.

**Supplementary Table S4. Extreme Quintile Analysis for Schizophrenia GPS**

| **SCZ GPS Quintile** | **Gestational age range** | **Mean cognitive score** | **SE Cognitive score** |
| --- | --- | --- | --- |
| Lowest quintile | Term (>=37 weeks) | 0.021 | 0.040 |
| Lowest quintile | <= 34 weeks | 0.089 | 0.070 |
| Highest quintile | Term (>= 37 weeks) | 0.004 | 0.040 |
| Highest quintile | <= 34 weeks | -0.258 | 0.077 |

**Supplementary Table S4.** Mean cognitive scores corrected for sex and SES and the associated standard errors (SE) for both term-born individuals and individuals born at or before 34 weeks completed gestation in the highest and lowest quintiles of the Schizophrenia GPS distribution.

**Supplementary Table S5. Extreme Quintile Analysis for Bipolar Disorder GPS**

| **BIP GPS Quintile** | **Gestation** | **Mean Cognitive score** | **SE Cognitive score** |
| --- | --- | --- | --- |
| Lowest quintile | Term (>=37 weeks) | 0.003 | 0.039 |
| Lowest quintile | <= 34 weeks | -0.025 | 0.069 |
| Highest quintile | Term (>= 37 weeks) | 0.060 | 0.037 |
| Highest quintile | <= 34 weeks | -0.106 | 0.085 |

**Supplementary Table S5.** Mean cognitive scores corrected for sex and SES and the associated standard errors (SE) for both term-born individuals and individuals born at or before 34 weeks completed gestation in the highest and lowest quintiles of the Bipolar Disorder GPS distribution.

**Supplementary Table S6. ANOVA analysis exploring a GxE interaction effect between psychiatric genetic risk and gestational age at birth for the five psychiatric pathologies.**

| **Psychiatric Pathology** | **N** | **F** | **p-value** |
| --- | --- | --- | --- |
| Schizophrenia | 1512 | 8.034 (df = 5, 1506) | 4.65x10^-3^ |
| Bipolar Disorder | 1536 | 1.551 (df = 5, 1530) | 0.213 |
| ASD | 1498 | 0.005 (df = 5, 1492) | 0.946 |
| ADHD | 1515 | 0.239 (df = 5, 1509) | 0.625 |
| Major Depressive Disorder | 1505 | 0.073 (df = 5, 1499) | 0.787 |

**Supplementary Table S6.** Results of two-way ANOVA analysis looking for a Gene x Environment interaction effect between psychiatric genetic risk and gestational age at birth. The analysis compares the mean cognitive scores at age 4 corrected for sex and socio-economic status for both term-born individuals and individual born at <=34 weeks in the highest and lowest quintiles of the polygenic risk score distributions. This table details the number of individuals (N), F-statistic (F) and associated p-values for each of the five psychiatric pathologies. Results for both Schizophrenia and Bipolar Disorder are presented in more detail in the main manuscript.

**Supplementary Tables S7a and S7b. ANOVA analysis exploring a GxE interaction effect between Schizophrenia genetic risk and gestational age at birth for a range of different gestational age cut-offs.**

| **Gestational age cut-offs (weeks)** | **Number of individuals** | **F-statistic** | **p-value** |
| --- | --- | --- | --- |
| >=37 and <=35 | 1704 | 5.791 (df = 5, 1698) | 0.016 |
| >=37 and <=34 | 1512 | 8.034 (df = 5, 1506) | 4.65 x 10^-3^ |
| >=37 and <=33 | 1363 | 6.703 (df = 5, 1357) | 9.73x10^-3^ |
| >=37 and <=32 | 1297 | 4.403 (df = 5; 1291) | 0.036 |

**Table 7a** Two-way ANOVA analysis comparing the mean cognitive scores at age four, corrected for both sex and socio-economic status for both term-born individuals (>= 37 weeks) and preterm individuals at a range of different gestational age cut-offs in the highest and lowest quintiles of the Schizophrenia GPS distribution.

| **Gestational age cut-offs (weeks)** | **Number of individuals** | **F-statistic** | **p-value** |
| --- | --- | --- | --- |
| >=37 and < 37 | 1974 | 1.348 (df = 5, 1968) | 0.246 |
| >=36 and <36 | 1974 | 8.048 (df = 5, 1968) | 4.60x10^-3^ |
| >=35 and <35 | 1974 | 9.709 (df = 5, 1968) | 1.86x10^-3^ |
| >=34 and <34 | 1974 | 7.034 (df = 5, 1968) | 8.06x10^-3^ |
| >=33 and <33 | 1974 | 4.309 (df = 5, 1968) | 0.038 |
| >=32 and <32 | 1974 | 2.969 (df = 5, 1968) | 0.085 |

**Table 7b** Two-way ANOVA analysis comparing the mean cognitive scores at age four, corrected for both sex and socio-economic status for individuals above and below a range of different gestational age cut-offs (from 37 to 32 weeks) in the highest and lowest quintiles of the Schizophrenia GPS distribution.

**Supplementary Table S8. Pearson correlation tests between the five psychiatric genome-wide polygenic scores investigated in this study.**

|  | **ASD** | **ADHD** | **Bipolar Disorder** | **Major Depressive Disorder** | **Schizophrenia** |
| --- | --- | --- | --- | --- | --- |
| **ASD** | 1 | < 2.2 x 10^-16^ | 0.0248 | < 2.2 x 10^-16^ | 4.24 x 10^-5^ |
| **ADHD** | 0.296 | 1 | 4.55 x 10^-3^ | < 2.2 x 10^-16^ | 2.32 x 10^-4^ |
| **Bipolar Disorder** | 0.032 | 0.040 | 1 | < 2.2 x 10^-16^ | < 2.2 x 10^-16^ |
| **Major Depressive Disorder** | 0.208 | 0.231 | 0.128 | 1 | < 2.2 x 10^-16^ |
| **Schizophrenia** | 0.058 | 0.052 | 0.266 | 0.197 | 1 |

**Supplementary Table S8***.*  Results from Pearson correlation tests between the five psychiatric genome-wide polygenic scores for the 4934 individuals investigated in this study. Values in the bottom diagonal represent Pearson correlation coefficients and values in the upper diagonal represent the corresponding p-values.

**Supplementary Table S9. Results from the final model generated using the stepwise linear regression model.**

| **Psychiatric Genetic Risk Interaction term** | ***β*** | **SE** | **p-value** |
| --- | --- | --- | --- |
| GPS_SCZ_ x GA | 0.029 | 0.015 | 0.048 |
| GPS_Bipolar_ x GA | 0.030 | 0.015 | 0.041 |

**Supplementary Table S9.** Results for the interaction terms between psychiatric genetic risk and gestational age are shown for the final model generated using stepwise linear regression. ﻿Stepwise linear regression was used to determine if both Schizophrenia and Bipolar Disorder interaction terms had an independent contribution in the regression model for cognition. Results are shown for the estimate of the effect size of the interaction terms in the final model (*β),* the standard error of the effect size estimate (SE) and the corresponding p-values.

**Supplementary Table S10. Association between genetic risk for psychiatric disease and cognitive outcome at age four.**

|  | ***GPS_psych_*** | | |
| --- | --- | --- | --- |
| **Psychiatric GPS** | ***β_1_*** | ***SE*** | ***p_1_*** |
| ADHD | -0.001 | 0.014 | 0.96 |
| Autism | 0.007 | 0.014 | 0.614 |
| Bipolar Disorder | 0.001 | 0.014 | 0.972 |
| MDD | -0.023 | 0.014 | 0.094 |
| Schizophrenia | -0.017 | 0.014 | 0.219 |

**Supplementary Table S10**. Results exploring the possible association between genetic risk for psychiatric disease (GPS_psych_) and cognitive outcome in 4934 unrelated individuals including sex and socio-economic status as covariates. *β_1_* is the estimate of the effect size for the psychiatric risk score, *SE* is the standard error of the effect size estimate and *p_1_* is the corresponding p-value.

**Supplementary Table S11. Association between genetic risk for psychiatric disease and gestational age at birth.**

|  | ***GA*** | | |
| --- | --- | --- | --- |
| **Psychiatric GPS** | ***β_1_*** | ***SE*** | ***p_1_*** |
| ADHD | -0.028 | 0.014 | 0.055 |
| Autism | -0.012 | 0.014 | 0.384 |
| Bipolar Disorder | 0.004 | 0.014 | 0.795 |
| MDD | -0.009 | 0.014 | 0.519 |
| Schizophrenia | 0.017 | 0.014 | 0.224 |

**Supplementary Table S11.** Results exploring the possible association between genetic risk for psychiatric disease (GPS_psych_) and gestational age at birth in 4934 unrelated individuals including sex as a covariate. *β_1_* is the estimate of the effect size of gestational age (GA), *SE* is the standard error of the effect size estimate and *p_1_* is the corresponding p-value.

**Supplementary Information References**

1. Keller, M. C. Gene × environment interaction studies have not properly controlled for potential confounders: The problem and the (simple) solution. *Biol. Psychiatry* **75**, 18–24 (2014).

2. Demontis, D. *et al.* Discovery of the first genome-wide significant risk loci for attention deficit/hyperactivity disorder. *Nat. Genet.* **51**, 63–75 (2019).

3. Grove, J. *et al.* Identification of common genetic risk variants for autism spectrum disorder. *Nat. Genet.* **51**, 431–444 (2019).

4. Sklar, P. *et al.* Large-scale genome-wide association analysis of bipolar disorder identifies a new susceptibility locus near ODZ4. *Nat. Genet.* **43**, 977–985 (2011).

5. Wray, N. R. *et al.* Genome-wide association analyses identify 44 risk variants and refine the genetic architecture of major depression. *Nat. Genet.* **50**, 668–681 (2018).

6. Pardiñas, A. F. *et al.* Common schizophrenia alleles are enriched in mutation-intolerant genes and in regions under strong background selection. *Nat. Genet.* **50**, 381–389 (2018).
